# ERβ1 Sensitizes and ERβ2 Desensitizes ERα-Positive Breast Cancer Cells to the Inhibitory Effects of Tamoxifen, Fulvestrant and Their Combination with All-Trans Retinoic Acid

**DOI:** 10.3390/ijms24043747

**Published:** 2023-02-13

**Authors:** Aggeliki K. Meligova, Dimitra Siakouli, Sotiria Stasinopoulou, Despoina S. Xenopoulou, Maria Zoumpouli, Vassiliki Ganou, Eleni-Fani Gkotsi, Aristotelis Chatziioannou, Olga Papadodima, Eleftherios Pilalis, Michael N. Alexis, Dimitra J. Mitsiou

**Affiliations:** 1 Institute of Chemical Biology, National Hellenic Research Foundation, 11635 Athens, Greece; 2 Center of Systems Biology, Biomedical Research Foundation of the Academy of Athens, 11527 Athens, Greece; 3 e-NIOS Applications PC, 25 Alexandros Pantou str., 17671 Kallithea, Greece

**Keywords:** breast cancer, ERβ1, ERβ2, prognostic markers, tamoxifen, ICI182,780, ATRA, gene expression, cell proliferation, cell death

## Abstract

Adjuvant endocrine therapy (AET) is the treatment of choice for early-stage estrogen receptor alpha (ERα)-positive breast cancer (BC). However, almost 40% of tamoxifen-treated cases display no response or a partial response to AET, thus increasing the need for new treatment options and strong predictors of the therapeutic response of patients at high risk of relapse. In addition to ERα, BC research has focused on ERβ1 and ERβ2 (isoforms of ERβ), the second ER isotype. At present, the impact of ERβ isoforms on ERα-positive BC prognosis and treatment remains elusive. In the present study, we established clones of MCF7 cells constitutively expressing human ERβ1 or ERβ2 and investigated their role in the response of MCF7 cells to antiestrogens [4-hydroxytamoxifen (OHΤ) and fulvestrant (ICI182,780)] and retinoids [all-trans retinoic acid (ATRA)]. We show that, compared to MCF7 cells, MCF7-ERβ1 and MCF7-ERβ2 cells were sensitized and desensitized, respectively, to the antiproliferative effect of the antiestrogens, ATRA and their combination and to the cytocidal effect of the combination of OHT and ATRA. Analysis of the global transcriptional changes upon OHT–ATRA combinatorial treatment revealed uniquely regulated genes associated with anticancer effects in MCF7-ERβ1 cells and cancer-promoting effects in MCF7-ERβ2 cells. Our data are favorable to ERβ1 being a marker of responsiveness and ERβ2 being a marker of resistance of MCF7 cells to antiestrogens alone and in combination with ATRA.

## 1. Introduction

Depending on expression of estrogen receptor alpha (ERα), progesterone receptor (PR) and human epidermal growth factor receptor 2 (HER2), breast cancer (BC) is classified as ERα-positive (ERα+/PR+/HER2±), EGFR2-overexpressing (ERα-/PR-/HER2+) and triple negative (ERα-/PR-/HER2-), with ≈70% of all BC cases categorized as ERα-positive [1]. ERα-positive BC is treated with adjuvant endocrine therapy (AET), which comprises selective ERα modulators (SERMs, e.g., tamoxifen), selective ERα degraders (SERDs, e.g., ICI182,780) and aromatase inhibitors (AIs, e.g., anastrazole), with tamoxifen for five years considered a treatment of choice for a large fraction of early-stage ERα-positive BC cases [2]. However, approx. 40% of tamoxifen-treated ERα-positive BC cases display no response or a partial response to AET, and disease recurrence 20 years after onset of AET following primary surgery is a fairly common outcome [3]. Since the recurrent disease is frequently more aggressive and/or metastatic, new treatment options and relevant strong predictors of the therapeutic response of those patients at intermediate or high risk of relapse are urgently needed. 

Retinoids, such as all-trans retinoic acid (ATRA, the major active form of vitamin A), inhibit proliferation and viability of tamoxifen-sensitive and tamoxifen-resistant ERα-positive BC cells and the growth of experimental ERα-positive breast tumors, but they reportedly have no effect on the survival of breast cancer patients whether administered alone or in combination with tamoxifen [4,5]. The anticancer activity of retinoids is mediated by three retinoic acid receptor isotypes (RARα, RARβ and RARγ) and three retinoid X receptor isotypes (RXRα, RXRβ and RXRγ) [6]. ATRA, in particular, is known to preferentially bind RARs and stimulate formation of RAR–RXR heterodimers, which bind to retinoic acid response elements (RAREs) of retinoid target genes to regulate gene expression [6]. RARα is preferentially expressed in ERα-positive BC cells, including MCF7 cells, a classical model of early-stage ERα-positive BC. In these cells, estrogen-stimulated ERα is known to induce RARα, which then can cooperate with chromatin-bound ERα to regulate estrogen-dependent cell proliferation and gene expression in concertation with other ERα cofactors [7]. Conversely, ATRA-stimulated RARα inhibits ERα-dependent cell proliferation and gene expression by competing with ERα for binding to adjacent or overlapping DNA regulatory elements and/or by interfering with ERα cofactor recruitment [7,8,9]. ATRA interference with ERα transcription complexes downregulates the expression of ERα and several estrogen-responsive genes, including PR and pS2, and in combination with 4-hydroxytamoxifen (OHT), an active metabolite of tamoxifen, synergistically inhibits proliferation of MCF7 cells [10,11,12]. It has been reported that ATRA treatment inhibits proliferation and viability of MCF7 cells by decreasing IGF-1 expression and secretion and by downregulating EGFR signaling and IRS-1 expression and signaling through the IRS-1/PI3kinase/AKT pathway [13,14,15,16]. 

OHT is known to act as estrogen agonist or antagonist through ERα, depending on cell-specific recruitment of coactivators or corepressors to genes that regulate key cell responses to the drug, including inhibition of cell proliferation [17,18]. It has been reported that in MCF7 cells, OHT upregulates the expression of a set of genes marginally induced by estrogen, including genes overexpressed in ERα-positive tumors of patients that suffered tumor relapse following primary surgery and adjuvant treatment with tamoxifen. Moreover, it was observed that introduction of ERβ1, the second ER isotype, inhibited OHT-induced upregulation of these genes in MCF7 cells and modulated the expression of unique as well as ERα-regulated genes in a manner that favors inhibition of cell proliferation and/or induction of apoptosis [19,20]. There are five isoforms of ERβ (ERβ1-5), of which only ERβ1, the product of the full transcript of ERβ gene, has high affinity for estradiol and tamoxifen [21,22]. ERβ1 and ERβ2 are the isoforms most frequently expressed in BC [23]. ERβ2 can modulate gene expression independently and/or in association with ERα, ERβ1 or other transcription factors [22,24,25,26]. ERβ1 is associated with an improved response of ERα-positive BC to tamoxifen, although not in all clinical studies [27,28]. We have previously reported that ERβ2 is associated with poor prognosis in ERα-negative BC and with higher risk of late relapse of AΕΤ-treated early-stage ERα-positive BC, while ERβ1 is associated with lower risk of early relapse of AET-treated early-stage ERα-positive BC [29,30]. In the present report, we compare the effects of OHT and ICI182,780 (ICI) used separately or in combination with ATRA on proliferation, viability and gene expression of wild-type MCF7 cells and clones thereof made to stably express ERβ1 or ERβ2. We show that ATRA potentiated the inhibitory effect of OHT and ICI on the proliferation and viability of wild-type MCF7 cells and that ERβ1 sensitized and ERβ2 desensitized the cells to inhibitory effects of OHT and ICI. Global gene expression analysis identified gene expression patterns associated with the response of ERβ1- or ERβ2-expressing MCF7 cells to the combination of OHT with ATRA, which suggest that ERβ1 and ERβ2 could function as predictors of therapeutic and adverse responses, respectively, of early-stage ERα-positive BC to antiestrogens alone or in combination with ATRA.

## 2. Results

### 2.1. Establishment of MCF7 Clones Stably Expressing Human ERβ1 or ERβ2 and Assessment of Their Effect on ERα Gene Expression and Transcriptional Activity

**Figure 1:**
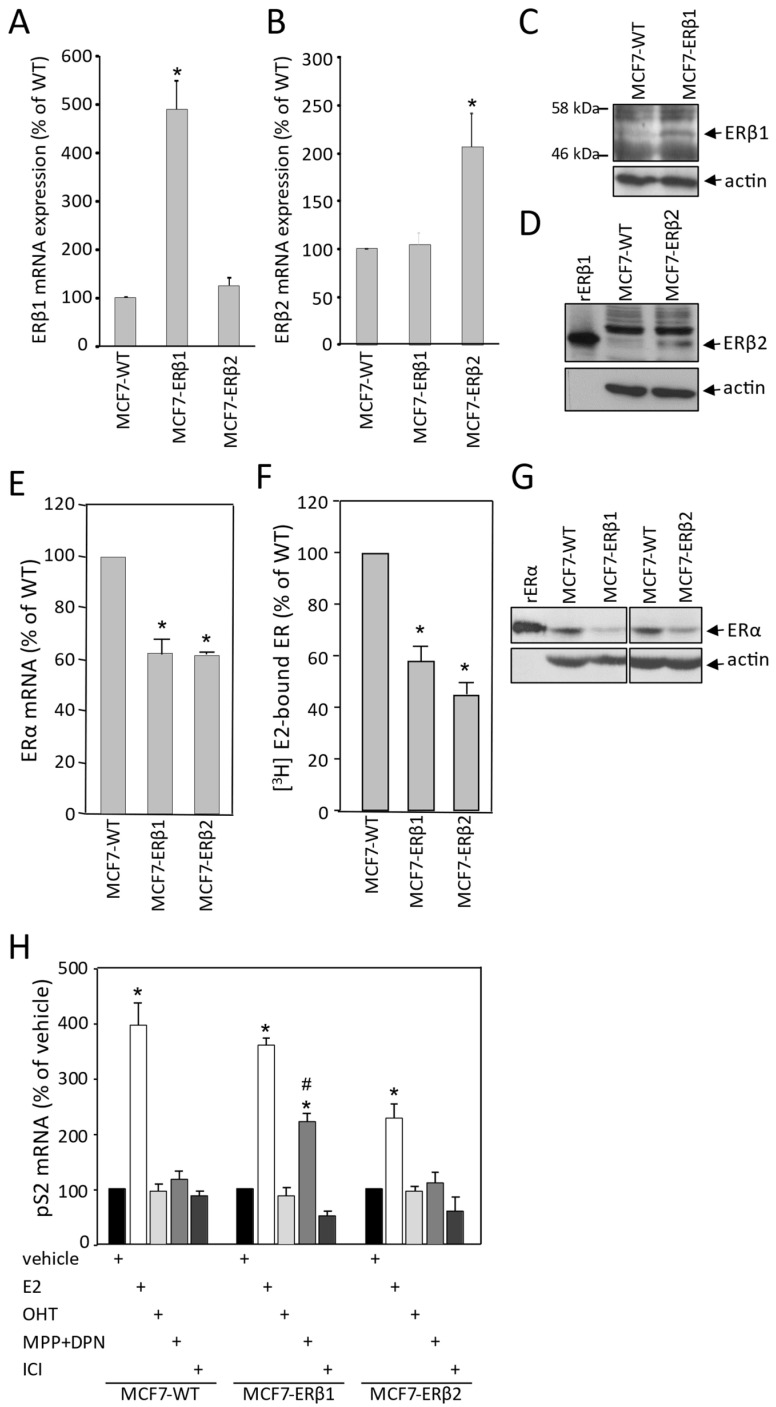
Establishment of clones of MCF7 cells stably expressing human ERβ1 or ERβ2. (**A**,**B**) mRNA levels of ERβ1 (**A**) and ERβ2 (**B**) in MCF7-ERβ1 and MCF7-ERβ2 cells were assessed by RT-qPCR and expressed relative to the levels in MCF7-WT cells. Values are mean ± SEM of at least three independent experiments carried out in triplicate. * *p* < 0.05 vs. WT cells (*t*-test). (**C**,**D**) Protein levels of ERβ1 in whole cell extracts from MCF7-WT and MCF7-ERβ1 (**C**) and ERβ2 in whole cell extracts from MCF7-WT and MCF7-ERβ2 cells (**D**) as assessed by Western blot analysis using antibodies against ERβ1, ERβ2 and actin (loading control). (**E**–**G**) ERβ1 or ERβ2 expression reduced ERα expression levels in MCF7 cells. (**E**) mRNA levels of ERα in MCF7-WT, MCF7-ERβ1 and MCF7-ERβ2 cells were assessed by RT-qPCR and expressed relative to the levels in MCF7-WT cells. (**F**) Levels of ER competent in high-affinity [3H]E2 binding were assessed in MCF7-WT, MCF7-ERβ1 and MCF7-ERβ2 cells by whole cell binding assays. Values are mean ± SEM of at least three independent experiments carried out in triplicate. * *p* < 0.05 vs. WT cells (*t*-test). (**G**) Protein levels of ERα in whole cell extracts from MCF7-WT, MCF7-ERβ1 and MCF7-ERβ2 cells as assessed with Western blot analysis using antibodies against ERα and actin (loading control). (**H**) ERβ1 is transcriptionally active in MCF7 cells: mRNA levels of pS2 gene in MCF7-WT, MCF7-ERβ1 and MCF7-ERβ2 cells, cultured in steroid-free medium and treated for 24 h with vehicle (0.1% DMSO), E2 (10 nM) or 100 nM of OHT, MPP+DPN or ICI were assessed by RT-qPCR and expressed relative to the respective levels upon treatment with vehicle alone. Values are mean ± SEM of at least three independent experiments carried out in triplicate. * *p* < 0.05 vs. vehicle of the respective cell line, ^#^ *p* < 0.05 vs. similarly treated MCF7-WT cells (*t*-test). OHT, 4-hydroxytamoxifen; MPP, methyl-piperidino-pyrazole; DPN, diarylpropionitrile; ICI, ICI182,780.

In order to investigate the role of ERβ isoforms in the response of breast cancer cells to antiestrogen treatment, we established clones of MCF7 cells constitutively expressing human ERβ1 or ERβ2. After initial screening, two clones were selected to be further used in the present study. Figure 1A,B show significantly higher specific expression of ERβ1 and ERβ2 mRNA in the MCF7-ERβ1 and the MCF7-ERβ2 cells, respectively. Western blot analysis confirmed the expression of ERβ1 and ΕRβ2 isoforms in MCF7-ERβ1 and MCF7-ERβ2 cells, respectively (Figure 1C,D). Next, we assessed the effect of ERβ1 and ERβ2 expression on ERα expression levels. ERα mRNA levels were significantly decreased in both MCF7-ERβ1 and MCF7-ERβ2 cells as compared to MCF7-WT cells (Figure 1E). Similarly, ERβ1- and ERβ2-expressing MCF7 cells displayed lower estrogen binding capacity as compared to WT cells (Figure 1F). In addition, ERα protein levels in both MCF7-ERβ1 and MCF7-ERβ2 were reduced as compared to MCF7-WT cells (Figure 1G). Taken together, our data demonstrate that expression of either ERβ1 or ERβ2 downregulates ERα expression in MCF7 cells.

To further extend our observations, we addressed the effect of ERβ1 and ERβ2 expression in MCF7 cells on the transcriptional regulation of the pS2 gene, an ERE-dependent estrogen target [31]. pS2 mRNA levels were significantly induced by estradiol in MCF7-WT and MCF7-ERβ1 cells (≈4.0- and 3.6-fold, respectively), whereas less pronounced induction (2.3-fold) was observed in cells expressing ERβ2 (Figure 1H). Expression of pS2 mRNA was not induced upon treatment with OHT and ICI. Importantly, significant induction of pS2 mRNA was observed upon treatment of MCF7-ERβ1 cells with a combination of the ERα-specific antagonist MPP [32] and the ERβ1-specific agonist DPN [33], suggesting that ERβ1 is transcriptionally active in the absence of ERα activation. 

### 2.2. ERβ1 Sensitized and ERβ2 Desensitized, Respectively, MCF7 Cells to the Antiproliferative Effect of OHT or ICI, Whether Used Separately or in Combination with ATRA

**Figure 2:**
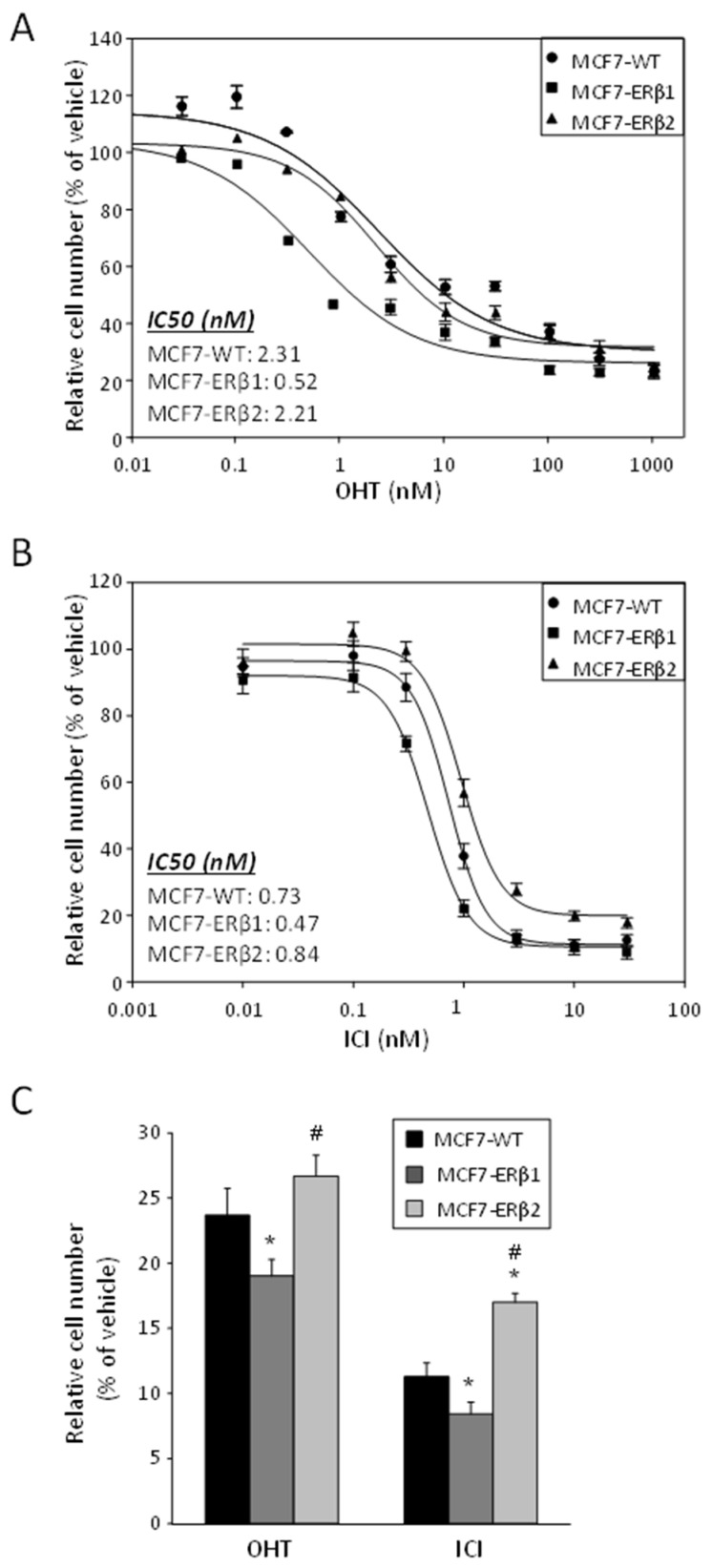
ERβ1 and ERβ2 inversely affect the antiproliferative efficacy of OHT and ICI. (**A**,**B**) Proliferation of MCF7-WT, MCF7-ERβ1 and MCF7-ERβ2 cells treated with vehicle (0.1% DMSO), increasing concentrations of OHT (**A**) or increasing concentrations of ICI182,780 (**B**) for six days. Relative cell numbers were expressed as percentage of the cell number of vehicle-treated cells. Plots are representative of at least three independent experiments carried out in triplicate. (**C**) Proliferation of MCF7-WT, MCF7-ERβ1 and MCF7-ERβ2 cells treated with vehicle (0.1% DMSO), ICI (30 nM) or OHΤ (300 nM) for six days. Relative cell numbers were expressed as percentage of cell number of vehicle-treated cells. Values are mean ± SEM of at least three independent experiments carried out in triplicate. * *p* < 0.05 vs. MCF7-WT, ^#^ *p* < 0.05 vs. MCF7-ERβ1 (*t*-test). OHT, 4-hydroxytamoxifen; ICI, ICI182,780.

Given our previous work showing a clinically better response of ERβ1-expressing ERα-positive breast tumors to AET and, conversely, increased late recurrence rate of ERα/ERβ2-expressing tumors [30], we assessed the response of MCF7-ERβ1 and MCF7-ERβ2 cells to OHT and ICI. Figure 2A shows the dose–response effect of OHT on estradiol (E2)-dependent proliferation of MCF7-WT, MCF7-ERβ1 and MCF7-ERβ2 cells. Interestingly, OHT inhibited proliferation of MCF7-ERβ1 cells with significantly higher potency and efficacy than that of MCF7-WT and MCF7-ERβ2 cells (Table 1), demonstrating that ERβ1 sensitized MCF7 cells to OHT. Considering that the maximum serum concentration (Cmax) of OHT is 0.11 μΜ [34], full suppression of cell proliferation at the Cmax is apparently possible for MCF7-ERβ1 cells but not for MCF7-WT cells or MCF7-ERβ2 cells (Figure 2A). The dose–response effect of ICI on the E2-dependent proliferation of MCF7-WT, MCF7-ERβ1 and MCF7-ERβ2 cells revealed that ICI inhibited proliferation of ERβ1- and ERβ2-expressing cells with significantly higher and lower potency, respectively, than that of WT cells (Figure 2B and Table 1), demonstrating that ERβ1 sensitizes and ERβ2 desensitizes MCF7 cells to ICI. Additionally, the efficacy of the antiproliferative effect of ICI was explicitly lower in ERβ2-expressing cells as compared to MCF7-WT cells. Full suppression of cell proliferation at the Cmax of ICI (0.04 μΜ; [34]) was apparently possible for WT and ERβ1-expressing cells but not for ERβ2-expressing cells (Figure 2B). The antiproliferative effect of pharmacologically relevant concentrations of ICI (30 nM) and OHT (300 nM) was further investigated. Significantly higher efficacy of OHT was observed for MCF7-ERβ1 cells, and tentatively lower efficacy was observed for MCF7-ERβ2 cells as compared to MCF7-WT cells (Figure 2C). In addition, ICI inhibited proliferation of all cells lines more effectively than OHT, with the efficacy for MCF7-ERβ1 and MCF7-ERβ2 cells being significantly higher and lower, respectively, as compared to MCF7-WT cells.

**Figure 3:**
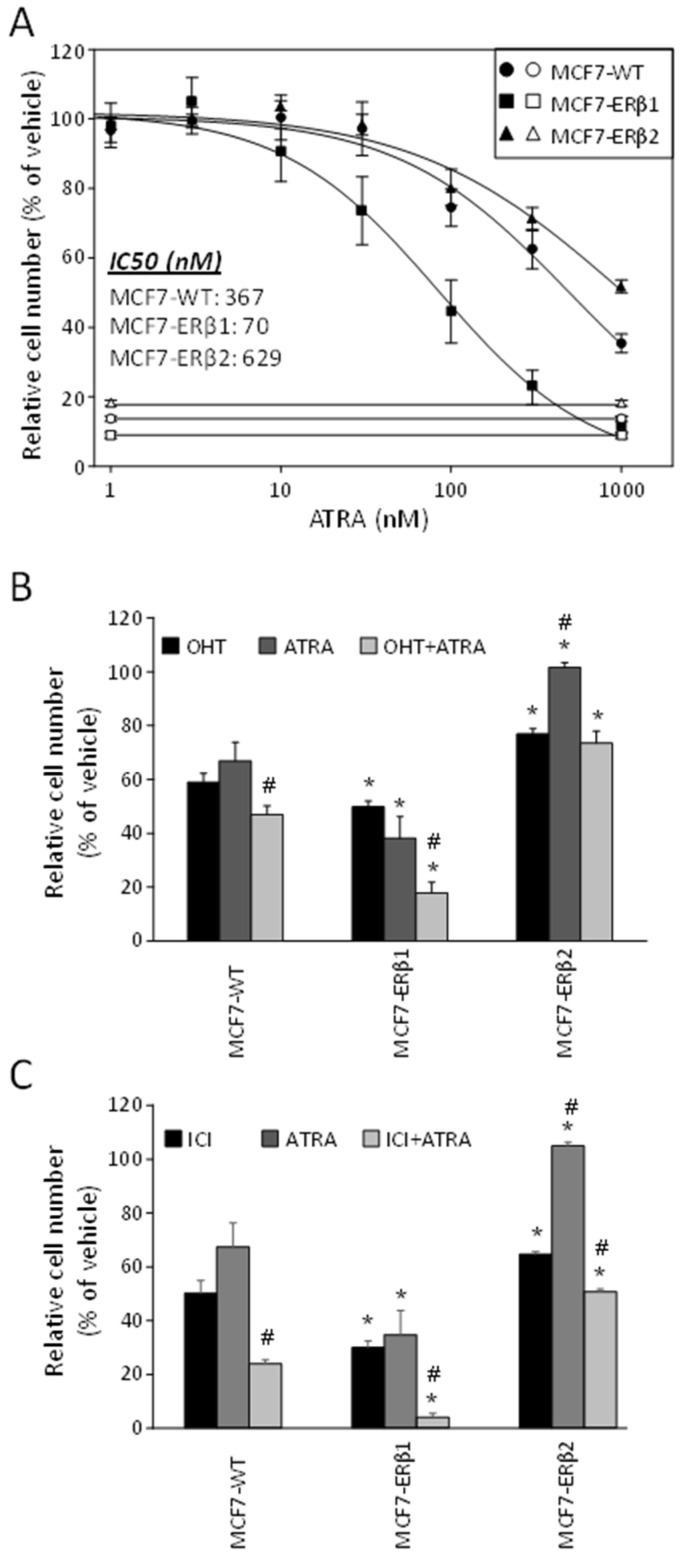
ERβ1 and ERβ2 inversely affect the antiproliferative efficacy of ATRA. (**A**) Proliferation of MCF7-WT, MCF7-ERβ1 and MCF7-ERβ2 cells treated with vehicle (0.1% DMSO) or increasing concentrations of ATRA for six days. Relative cell numbers were expressed as percentage of cell number of vehicle-treated cells. Plots are representative of at least three independent experiments carried out in triplicate. (**B**,**C**) Proliferation of MCF7-WT, MCF7-ERβ1 and MCF7-ERβ2 cells treated with vehicle (0.1% DMSO), OHT (1 nM), ICI (1 nM), ATRA (100 nM) or their combination, as indicated, for six days. Relative cell numbers were expressed as percentage of cell number in vehicle-treated cells. Values (mean ± SEM) are from at least three independent experiments carried out in triplicate. * *p* < 0.05 vs. similarly treated MCF7-WT, ^#^ *p* < 0.05 vs. respective OHT- or ICI-treated cell line (*t*-test). OHT, 4-hydroxytamoxifen; ATRA, all-trans retinoic acid; ICI, ICI182,780.

To extend our data, we next investigated the effect of ERβ1 and ERβ2 expression on the response of MCF7 cells to ATRA. Increasing concentrations of ATRA reduced proliferation of MCF7-WT, MCF7-ERβ1 and MCF7-ERβ2 cells. Importantly, ATRA inhibited the E2-dependent proliferation of ERβ1- and ERβ2-expressing MCF7 cells with significantly higher and lower potency/efficacy, respectively, as compared to MCF7-WT cells (Figure 3A and Table 2). Considering that the maximum serum concentration (Cmax) of ATRA is 1.15 μΜ [34], full suppression of cell proliferation at Cmax of ATRA was apparently possible for MCF7-ERβ1 cells but not for MCF7-WT cells or MCF7-ERβ2 cells (Figure 3A).

Finally, in order to gain more insight into the joint effects of ATRA and antiestrogens on MCF7 cell proliferation, we investigated the antiproliferative effect of the combination of ATRA (100 nM) with OHT (1 nM) or ICI (1 nM). The selected concentrations for each compound alone caused partial inhibition of E2-dependent cell proliferation (Figure 2A,B and Figure 3A). Figure 3B,C show that the antiproliferative efficacy of OHT+ATRA and ICI+ATRA significantly exceeded that of OHT, ICI and ATRA acting as single agents in MCF7-WT cells; that the antiproliferative efficacy of OHT, ICI, ATRA and the combinations of OHT+ATRA and ICI+ATRA was significantly higher in MCF7-ERβ1 cells compared to MCF7-WT cells; and that the antiproliferative efficacy of OHT+ATRA and ICI+ATRA in ERβ1-expressing cells significantly exceeded that of OHT, ICI and ATRA acting as single agents. In contrast, the antiproliferative efficacy of OHT, ICI, ATRA and their combinations was significantly lower in MCF7-ERβ2 cells compared to MCF7-WT cells. In addition, the efficacy of ATRA as single agent in ERβ2 cells was significantly lower than that of OHT, ICI and their combination with ATRA. Taken together, the above data show that ERβ1 increases and ERβ2 decreases the antiproliferative efficacy of ATRA, OHT+ATRA or ICI+ATRA in MCF7 cells.

### 2.3. OHT, ICI and ATRA Abolished the S-Phase Fraction of ERβ1-Expressing MCF7 Cells

**Figure 4:**
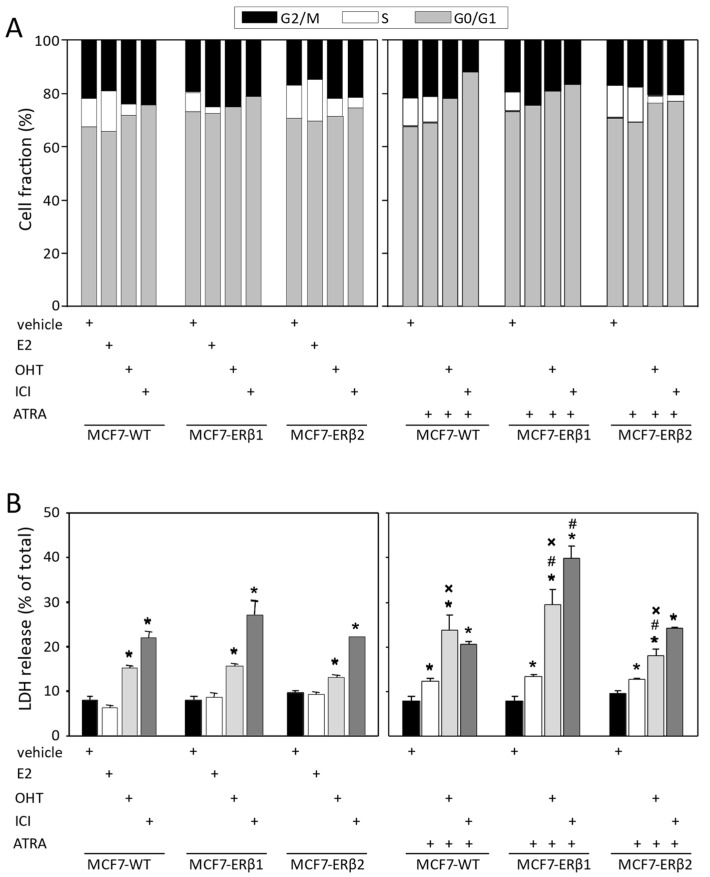
OHT, ICI and ATRA abolished the S-phase fraction and induced cell death in ERβ1-expressing cells. (**A**) Cell cycle phase distribution of MCF7-WT, MCF7-ERβ1 and MCF7-ERβ2 cells treated for 72 h with vehicle (0.1% DMSO), E2 (10 nM), OHT (100 nM), ICI (100 nM), ATRA (50 nM) or their indicated combination, as assessed by flow cytometry. Plots are representative of at least three independent experiments. (**B**) LDH release (relatively to total LDH) from MCF7-WT, MCF7-ERβ1 and MCF7-ERβ2 cells treated as in (**A**). Values are mean ± SEM of at least three independent experiments carried out in triplicate. * *p* < 0.05 vs. vehicle of the respective cell line, ^#^ *p* < 0.05 vs. similarly treated MCF7-WT cells, ^x^ *p* < 0.05 vs. single-agent treatment of the respective cell line. E2, 17β-estradiol; OHT, 4-hydroxytamoxifen; ATRA, all-trans retinoic acid; ICI, ICI182,780; LDH, lactate dehydrogenase.

Next, we investigated the effect of OHT, ICI, ATRA and their combinations on the cell cycle phase distribution of MCF7-WT, MCF7-ERβ1 and MCF7-ERβ2 cells as assessed by flow cytometry. E2 treatment resulted in a tentatively significant increase in the S-phase fraction of MCF7-WT, whereas it significantly reduced the S-phase fraction of MCF7-ERβ1 cells (Figure 4A and Supplementary Table S1). OHT treatment abolished the S-phase fraction of MCF7-ERβ1 cells and significantly reduced the S-phase fraction of MCF7-WT cells but not that of MCF7-ERβ2 cells. ICI treatment abolished the S-phase fraction of MCF7-WT and MCF7-ERβ1 cells and significantly reduced but failed to abolish that of MCF7-ERβ2 cells. On the other hand, ATRA alone abolished the S-phase fraction of MCF7-ERβ1 cells but did not significantly affect that of MCF7-WT or MCF7-ERβ2 cells. When in combination with OHT or ICI, ATRA eliminated the S-phase fraction of MCF7-WT and MCF7-ERβ1 cells and significantly reduced but not eliminated that of MCF7-ERβ2 cells (Figure 4A and Supplementary Table S1). In short, while the proliferation of ERβ1-expressing cells was fully inhibited by OHT at 100 nM (≈Cmax), ATRA at 50 nM (≈0.04 × Cmax) and their combination, the proliferation of wild-type cells was fully inhibited only by the combination, and the proliferation of ERβ2-expressing cells was inhibited by none of the three treatments. Moreover, in contrast to wild-type and ERβ1-expressing cells, the proliferation of ERβ2-expressing cells was not inhibited by ICI at 100 nM (=2.5 × Cmax) or its combination with 50 nM ATRA. Of note, the downsizing of the S-phase fraction induced by the combination of ICI and ATRA was accompanied by an increase in the G0/G1-phase fraction that was significant for MCF7-WT cells and tentatively significant for ERβ1- and ERβ2-expressing cells; however, a tentatively significant increase in the G2/M-phase fraction at the expense of S-phase fraction was observed following treatment of MCF7-ERβ2 cells with OHT and ICI (Figure 4A).

### 2.4. ERβ1 Enhanced the Cytocidal Effects of ATRA Combinations with OHT or ICI 

To gain insight into the mechanisms underlying the response of ERβ1- and ERβ2-expressing MCF7 cells to OHT, ICI, ATRA and their combinations, we tested whether these treatments induce cell death. Preliminary experiments revealed that the cell death observed following single-agent or combinatorial treatment was not apoptotic. We therefore resorted to quantifying non-apoptotic cell death by measuring LDH release (lactate dehydrogenase, an enzyme rapidly released in the cell culture supernatant when the plasma membrane is damaged). By taking the release of LDH as a measure of cell death, we show that OHT, ICI and ATRA induced cell death of MCF7-WT, MCF7-ERβ1 and MCF7-ERβ2 cells independently of one another, with ICI exerting the most pronounced effect (Figure 4B). Importantly, the combination of ATRA with OHT or ICI significantly increased cell death in ERβ1-expressing cells as compared to similarly treated MCF7-WT cells. In contrast, significantly decreased cell death of ERβ2-expressing cells, as compared to similarly treated MCF7-WT cells, was observed upon treatment with the combination of ATRA with OHT. Moreover, the combination of ATRA with OHT significantly increased cell death as compared to OHT alone in all cell lines.

**Figure 5:**
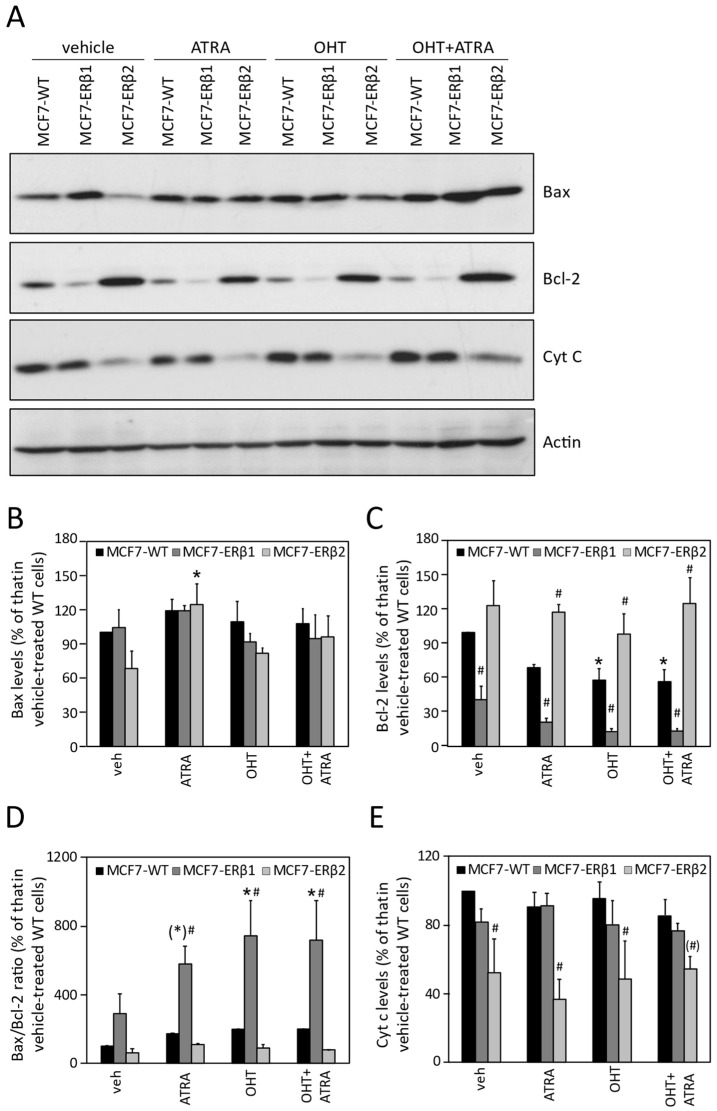
Modulation of Bax, Bcl-2 and cytochrome c expression by OHT, ATRA and their combination. (**A**) Western blot analysis of whole cell extracts from 2 × 10^5^ MCF7-WT, MCF7-ERβ1 and MCF7-ERβ2 cells treated for 24 h with vehicle (0.1% DMSO), OHT (100 nM), ATRA (50 nM) or their combination, as indicated. Antibodies against Bax, Bcl-2, cytochrome c and actin (loading control) were used. (**B**–**E**) Densitometric analysis of Bax (**B**), Bcl-2 (**C**) and cytochrome c protein bands. Density of Bax, Bcl-2 and cytochrome c protein bands was normalized to density of the respective actin bands and expressed relative to that of vehicle-treated MCF7-WT cells. The Bax/Bcl-2 ratio was calculated from the respective Bax and Bcl-2 band densities. * *p* < 0.05 and ^(*)^ *p* = 0.07 vs. vehicle of the respective cell line, ^#^ *p* < 0.05 and ^(#)^ *p* = 0.08 vs. similarly treated MCF7-WT cells. OHT, 4-hydroxytamoxifen; ATRA, all-trans retinoic acid.

To extend and corroborate the above data, we assessed the effect of OHT, ATRA and their combination on the levels of Bax (pro-apoptotic protein), Bcl-2 (anti-apoptotic protein) and cytochrome c (released from mitochondria to the cytoplasm in cells undergoing apoptosis). Notably, several reports in the literature suggest that expression of these apoptosis-related proteins is similarly modulated in non-apoptotic as well as in apoptotic cell death. Figure 5A depicts Bax, Bcl-2 and cytochrome c protein levels in MCF7-WT, MCF7-ERβ1 and MCF7-ERβ2 cells upon treatment with OHT, ATRA and OHT+ATRA. Densitometric analysis revealed that Bax expression was significantly increased only in MCF7-ERβ2 cells as compared to MCF7-WT cells upon treatment with ATRA (Figure 5B). Importantly, treatment with ATRA or OHT alone or with their combination significantly decreased Bcl-2 expression (Figure 5C) and increased the Bax-to-Bcl-2 expression ratio of MCF7-ERβ1 cells (Figure 5D). In contrast, all treatments resulted in significantly increased Bcl-2 expression and decreased cytochrome c expression in MCF7-ERβ2 cells compared to MCF7-WT cells (Figure 5C,E). Taken together, the modulation of Bax, Bcl-2 and cytochrome c expression by the combination of OHT with ATRA is consistent with increased cell death of ERβ1-expressing cells and decreased cell death of ERβ2-expressing cells compared to wild-type cells, as probed by LDH release.

### 2.5. Global Transcriptional Profile of MCF7-WT, MCF7-ERβ1 and MCF7-ERβ2 Cells Treated with OHT+AΤRA

**Figure 6:**
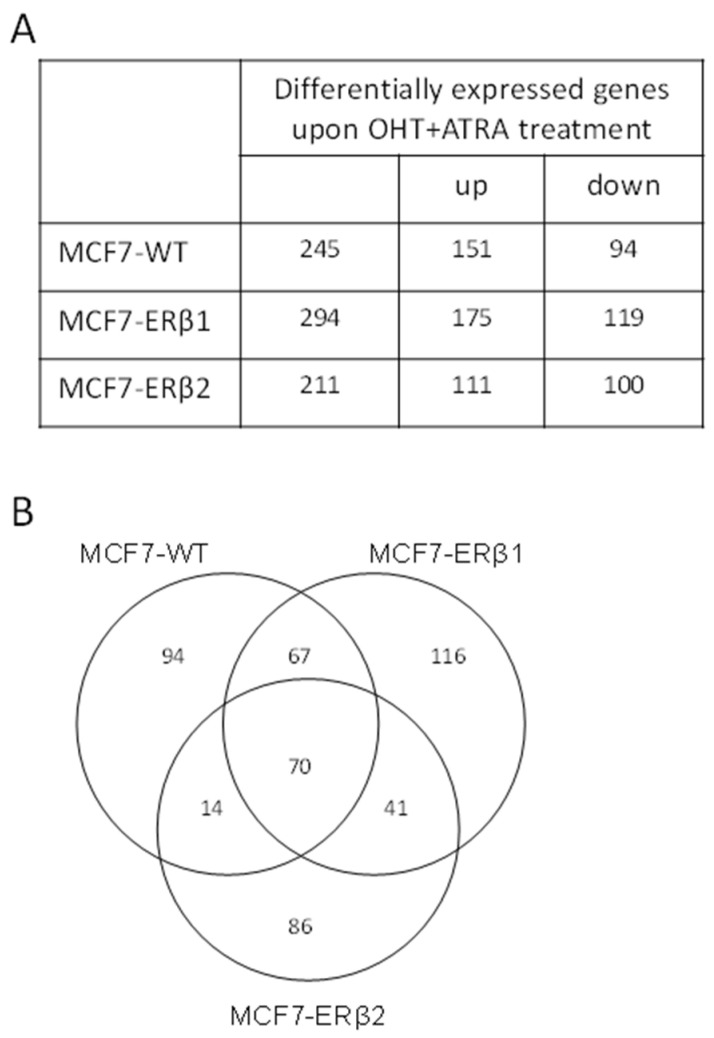
Global transcriptional profile of MCF7-WT, MCF7-ERβ1 and MCF7-ERβ2 cells in response to treatment with OHT in combination with ATRA. (**A**) The number of differentially expressed genes in MCF7-WT, MCF7-ERβ1 and MCF7-ERβ2 cells upon treatment with OHT (100 nM)+AΤRA (50 nM). (**B**) Venn diagram representing the overlap between differentially expressed genes in MCF7-WT, MCF7-ERβ1 and MCF7-ERβ2 cells. OHT, 4-hydroxytamoxifen; ATRA, all-trans retinoic acid.

To gain insight into the mechanisms underlying the response of ERβ1- and ERβ2-expressing MCF7 cells to the combination of OHT and AΤRA, we determined the global transcriptional changes induced upon treatment of MCF7-WT, MCF7-ERβ1 and MCF7-ERβ2 cells with OHT+AΤRA by performing a microarray-based differential gene expression analysis. Our data revealed 245, 294 and 211 differentially expressed genes, as compared to vehicle treatment, in MCF7-WT, MCF7-ERβ1 and MCF7-ERβ2 cells, respectively (Figure 6A and Supplementary Tables S2–S4). The microarray data were validated by real time PCR of 10 randomly selected genes differentially expressed in MCF7-WT, MCF7-ERβ1 and MCF7-ERβ2 cells. Supplementary Figure S1 shows that the relative expression levels of the selected genes as computed by the two methodologies are strongly correlated. Comparison of the repertoires of differentially expressed genes showed that approximately 40% are unique for each cell line (Figure 6B), indicating that OHT+AΤRA treatment alters unique as well as common signaling pathways.

**Figure 7:**
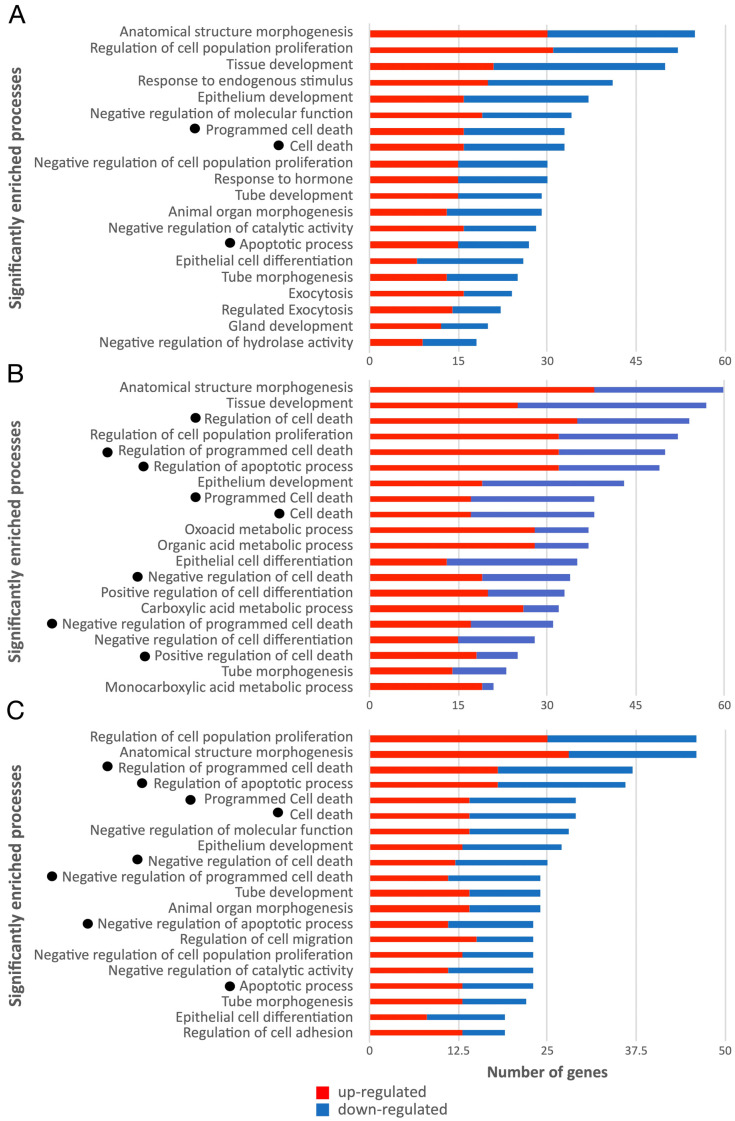
Enrichment analysis of biological processes related to genes regulated by OHT in combination with ATRA. Statistically significantly enriched biological processes based on Gene Ontology enrichment analysis, with the number of corresponding genes, upon treatment of MCF7-WT (**A**) MCF7-ERβ1 (**B**) and MCF7-ERβ2 cells (**C**) with OHT in combination with ATRA. The number of significantly differentially expressed genes involved in each process is shown on the X axis, with red and blue colors corresponding to up- or downregulation, respectively. Cell-death-related processes are indicated with dots. OHT, 4-hydroxytamoxifen; ATRA, all-trans retinoic acid.

Functional analysis, based on Gene Ontology (GO) biological process enrichment, for each set of differentially expressed genes was performed. The top 20—in terms of the number of relevant genes—statistically significant biological processes identified for MCF7-WT, MCF7-ERβ1 and MCF7-ERβ2 cells are shown in Figure 7. The complete lists of the enriched GO terms are presented in Supplementary Tables S5–S7. With the scope of discriminating genes with a putatively central role in the cellular response to the applied treatment, topological analysis exploiting BioInfoMiner was performed, where semantic information is used to prioritize genes based on their centrality as described in knowledge networks such as the GO. This analysis resulted in a short list of genes (referred to as hub genes) for each cell line (Supplementary Table S8), which represent possible master regulators of the cellular response to the treatment with OHT+AΤRA. Interestingly, among the significantly enriched biological processes altered by OHT+AΤRA in MCF7-ERβ1 and MCF7-ERβ2 cells, there were eight cell-death-related processes, whereas in the case of MCF7-WT cells, only three cell-death-related processes were found. Collectively, 96 differentially expressed genes were involved in the identified cell-death-related processes in at least one cell line, with the corresponding genes being 35, 57 and 51 for MCF7-WT, MCF7-ERβ1 and MCF7-ERβ2 cells, respectively (Supplementary Table S9). Comparison of the expression profiles of the 96 cell-death-related genes revealed that 10 genes were commonly regulated in all cell lines, whereas 13, 28 and 18 genes were unique for MCF7-WT, MCF7-ERβ1 and MCF7-ERβ2 cells, respectively (Table 3, Table 4 and Table 5).

In MCF7-WT cells, the regulation of the two unique hub genes (BCL2 and NKX3-1) and of five more unique genes (S100A8, TNFRSF11B, KRT10, DRAM1 and IRF1) is reportedly associated with anticancer effects, while the regulation of the remaining six unique genes (CADM1, KRT4, SQSTM1, HK2, DHCR24 and RAI14) is reportedly associated with cancer-promoting effects [35,36,37,38,39,40,41,42,43,44,45,46,47]. By comparison, analysis of the role of the unique genes in MCF7-ERβ1 cells revealed that the regulation of all but one of the hub genes (PPARG, HIPK2, ZFP36L1, HMGB2 and ALDH1A3) and of 12 more unique genes (ASCL1, ID3, GPNMB, SGK3, BAG3, WDR73, ALKBH1, HIGD1A, FGD3, ACAA2, DOCK8 and AGR3) reportedly favors inhibition of breast cancer cell proliferation and tumorigenicity, induction of cell death and/or better clinical outcome [48,49,50,51,52,53,54,55,56,57,58,59,60,61,62,63,64]. In contrast, in ERβ2-expressing cells, the regulation of the two unique hub genes (CXCL12 and SIX4) and of nine of the other unique genes (INHBE, GADD45B, RPS7, IFIT3, IGFBP3, JAG2, BCL3, GPR37L1 and KRT80) reportedly favors inhibition of cell death, resistance of BC to endocrine therapy and poor clinical prognosis [65,66,67,68,69,70,71,72,73,74,75]. Indicative functions reported for all cell-death-related genes uniquely regulated in MCF7-WT, MCF7-ERβ1 and MCF7-ERβ2 cells are presented in Supplementary Tables S10–S12. The above data may be taken to suggest that the modulation of expression of uniquely regulated genes in ERβ1- and ERβ2-expressing MCF7 cells by OHT in combination with ATRA is in accordance with ERβ1 being a marker of responsiveness and ERβ2 being a marker of resistance of ERα-positive BC cells to the combinatorial treatment.

## 3. Discussion

In the present study, we investigated the role of ERβ isoforms in the response of breast cancer cells to antiestrogen and retinoid treatment. We established clones of MCF7 cells constitutively expressing human ERβ1 or ERβ2 and showed that ERβ1 and ERβ2 sensitizes and desensitizes, respectively, MCF7 cells to the antiproliferative effect of antiestrogens (OHT and ICI), retinoids (ATRA) and their combination. Combination of ATRA with OHT significantly increased and decreased cell death of MCF7-ERβ1 and MCF7-ERβ2 cells, respectively, compared to wild-type MCF7 cells. Analysis of the global transcriptional profile of these cells upon treatment with OHT+ATRA revealed uniquely regulated genes that are favorable to ERβ1 being a marker of responsiveness and ERβ2 being a marker of resistance of MCF7 cells to combinatorial treatment with OHT and ATRA.

The lower levels of ERα mRNA in MCF7-ERβ1 cells, as compared to WT cells, are in agreement with previous data reporting ERβ1-induced downregulation of ERα gene expression through the ERβ1–Sp1 interaction and NCoR corepressor recruitment on the ERα promoter [76]. In addition, ERE-dependent transrepression of ERα gene expression by the ERβ2/ERα heterodimer, as already reported [24], may account for the lower levels of ERα mRNA in MCF7-ERβ2 cells as compared to MCF7-WT cells. Transrepression by ERβ2 alone may be excluded considering that ERβ2 is by itself reportedly unable to modulate ERE-dependent gene transcription [77]. The lower ERα protein levels (that are able to bind estrogen with high affinity) in MCF7-ERβ2 cells may be, at least in part, due to ERβ2-mediated proteasome-dependent degradation of ERα [24]. 

The reduced induction of transcription of the pS2 gene, an ERE-dependent estrogen-inducible target [31], upon E2 treatment of MCF7-ERβ1 and MCF7-ERβ2 cells, as compared to MCF7-WT cells, may be due to the lower ERα levels in these cells. Reduced E2-mediated transcription of the pS2 gene in MCF7-ERβ2 cells, as compared to MCF7-WT and MCF7-ERβ1 cells, also indicates ERE-dependent transrepression of pS2 expression by the ERβ2/ERα heterodimer as already reported [24]. Induction of pS2 upon MPP+DPN treatment indicates that ERβ1 is transcriptionally active in the absence of ERα activation, which agrees with recent reports [78]. Transcriptional activation by ERβ1, either as an ERβ1/ERα heterodimer or ERβ1 homodimer, could account for the higher E2-mediated pS2 transcription in MCF7-ERβ1 as compared to MCF7-ERβ2 cells. 

We showed that OHT and ICI inhibited the E2-dependent proliferation of MCF7-ERβ1 cells with significantly higher potency and efficacy than that of MCF7-WT cells. This finding is in accordance with previous reports on the role of ERβ1 in enhancing the antiproliferative efficacy and potency of antiestrogens such as OHT and ICI [79]. Co-expression of ERα and ERβ1 appears to support formation of ERα/ERβ1 heterodimers of potentially higher antiproliferative potency, which is reportedly dependent on the ligand [80]. Co-expression of ERα and ERβ2 in MCF7-ERβ2 cells resulted in lower efficacy of OHT either due to repression of ERα-mediated transactivation and induction of proteasome-dependent degradation of ERα by ERβ2 (as already reported [24]) or due to ERα-independent induction of proliferation by ERβ2, as shown for MDA-MB-231 cells [81]. The significantly higher and lower efficacy of OHT in ERα/ERβ1- and ERα/ERβ2-expressing cells, respectively, compared to cells expressing only ERα could account for the clinically better response of ERβ1-expressing ERα-positive breast tumors to endocrine therapy and, conversely, for the increased recurrence rate of ERα/ERβ2-expressing tumors [30].

The lower antiproliferative potency and efficacy of ICI in ERβ1-expressing cells could be attributed to agonist effects on other modulators, such as the G protein-coupled estrogen receptor (GPER) [82], whereas in ERβ2-expressing cells, it may reflect an inherently higher capacity of ERβ2 to drive MCF7 cell proliferation in the absence of ICI-degraded ERα, as previously reported for MDA-MB-231 cells [81]. ICI is an ER destabilizer [83] that promotes proteasome-dependent degradation of ERα and ERβ1 [79]. ERβ2 could increase the basal rate of cell proliferation at pharmacologically relevant concentrations of ICI, which are capable of fully destabilizing ERα but are unable to affect expression and signaling of ERβ2 given that the latter is reportedly unable to bind ligands even at relatively high concentrations [22,84]. Persistent ERβ2 signaling could account for the late recurrence of ERα/ERβ2-expressing tumors [30]. The lower antiproliferative effect of ICI at the Cmax (0.040 μM) [34] in ERα/ERβ2-expressing cells compared to ERα/ERβ1 cells implies that Faslodex treatment of recurrent ERα-positive tumors that express ERβ2 is eventually bound to fail to prevent disease recurrence.

The observed inhibitory effect of ATRA on E2-dependent proliferation of MCF7 cells is in accordance with previous reports on the role of ATRA in promoting inhibition of cell proliferation and enhancement of cell death in MCF7 cells through various mechanisms [13,14,15,16,85]. The higher potency and efficacy of ATRA in MCF7-ERβ1 cells could be attributed to ATRA-stimulated RARα interference with cofactor recruitment to the ERβ1/ERα heterodimer resulting in enhanced downregulation of ERβ1/ERα-dependent cell proliferation as compared to the ERα/ERα-dependent one [7,8,9]. Induction of MCF7 cell proliferation by ERβ2, as already reported for MDA-MB-231 cells [81], may account for the lower potency and efficacy of ATRA in MCF7-ERβ2 cells.

It has been reported that the combination of ATRA and OHT suppressed proliferation and viability of MCF7 cells more than OHT [10,11]. We corroborated these findings and further showed that the combination of ATRA with either OHT or ICI suppressed proliferation and viability of MCF7-ERβ1 cells more effectively than those of MCF7-WT cells and that the combination of ATRA with either OHT or ICI suppressed proliferation of MCF7-ERβ2 cells less effectively than that of MCF7-WT cells. 

The decrease in the S-phase fraction of MCF7-ERβ1 cells upon E2 treatment, as compared to MCF7-WT cells, is in agreement with previous reports [79,86]. The difference with the recently reported E2-induced increase in the S-phase fraction in MCF7 cells expressing only ERβ1 [78] indicates that the presence of ERα drastically modifies ERβ1 signaling to the proliferation machinery of MCF7 cells. In fact, it has been shown that introduction of ERβ1 in MCF7 cells inhibited the expression of unique as well as ERα-regulated genes in a manner that favors inhibition of E2-dependent cell proliferation and/or induction of cell death [20]. The abolishment of the S-phase fraction in MCF7-ERβ1 cells compared to MCF7-WT cells upon OHT treatment coincides with an increase in G1- and G2/M-phase fractions at the expense of part of the S-phase fraction, as previously reported for OHT-treated ERβ1-expressing MCF7 cells [79]. Analysis of our data reveals that the effects of OHT/ICI and/or ATRA on cell cycle phase distributions of MCF7-WT, MCF7-ERβ1 and MCF7-ERβ2 cells correlate well with the effects of the respective treatments on cell proliferation and cell death. Notably, the combinations of ATRA with OHT or ICI that exerted the most pronounced antiproliferative and cell death effects in MCF7-ERβ1 cells completely abolished the S-phase fraction of these cells but not of MCF7-ERβ2 cells.

The observed induction of MCF7 cell death by OHT, ICI or ATRA has been previously reported [85,87]. The significantly increased and decreased cell death by the combination of ATRA with OHT in MCF7-ERβ1 and MCF7-ERβ2 cells, respectively, is consistent with the modulation of Bcl-2 levels, the Bax-to-Bcl-2 expression ratio and cytochrome c levels in these cells. The robust downregulation of Bcl-2 expression and increase in the Bax-to-Bcl-2 expression ratio in MCF7-ERβ1 cells as well as the downregulation of cytochrome c expression in MCF7-ERβ2 cells alludes to different mechanisms of cell death depending on ERβ1 and ERβ2 expression and to the presumption that ERβ1 is sensitive and ERβ2 insensitive to antiestrogens and the antiestrogen component of combinatorial treatments. Downregulation of Bcl-2 expression and an increased Bax-to-Bcl-2 expression ratio may be associated with non-apoptotic cell death in the antiestrogen-responsive MCF7-WT and MCF7-ERβ1 cells in agreement with the previously reported induction of non-apoptotic cell death upon treatment of MCF7 cells with OHT and ICI 164 384 [88] and suppression of the pro-survival role of Bcl-2 in ERα-positive breast cancer cells by antiestrogens [89]. In addition, decrease in both cytochrome c and LDH release in MCF7-ERβ2 cells may suggest suppression of non-apoptotic cell death in these cells in accordance with previously reported data showing that cytochrome c release precedes necrosis as well as apoptosis [90]. The apparently different molecular mechanisms of cell death of MCF7-ERβ1 and MCF7-ERβ2 cells warrant further investigation.

Analysis of global transcriptional changes in response to the combination of ATRA with OHT supports our observations regarding cell proliferation and cell death. Downregulation of BCL2 and upregulation of NKX-3, the two unique hub genes in MCF-WT cells, are both consistent with anticancer effects [35,36]. Concerning all the genes that are unique to MCF7-WT cells, the regulation of almost half of them is reportedly associated with anticancer effects, while the regulation of the other half is associated with cancer-promoting effects. Upregulation of five out of the six unique hub genes in MCF7-ERβ1 cells reportedly favors inhibition of breast cancer cell proliferation and tumorigenicity, induction of cell death and/or better clinical outcome [48,49,50,51,52], which is in accordance with the decreased proliferation and increased cell death observed in response to ATRA+OHT compared to OHT. In contrast, downregulation of CXCL12 and upregulation of SIX4, the two unique hub genes in MCF7-ERβ2 cells, are reportedly consistent with cancer-promoting effects and are linked to poor prognosis [65,66]. Regarding all unique genes, 61% of them as regulated in MCF7-ERβ1 and MCF7-ERβ2 cells are reportedly associated with anti-cancer and cancer-promoting effects, respectively. Our analysis revealed that the five unique hub genes (PPARG, HIPK2, ZFP36L1, HMGB2 and ALDH1A3) may constitute a gene expression signature specifying a therapeutic response of ERβ1-expressing ERα-positive breast cancer cells to treatment with OHT+ATRA.

## 4. Materials and Methods

### 4.1. Purchased Compounds, Chemicals and Plasmids

17β-estradiol (E2), 4-OH tamoxifen (OHT), all-trans retinoic acid (ATRA) and geneticin (G418) were purchased from Sigma-Aldrich (St Louis, MO, USA). ICI 182,780 (ICI), methyl-piperidino-pyrazole (MPP) and diarylpropionitrile (DPN) were obtained from Tocris Bioscience (Bristol, UK). [3H]E2 (1 μCi/μL, 83.0 Ci/mmol) was obtained from Amersham Biosciences. Plasmids pcDNA3.1-hERβ1, pcDNA3.1-hERβ2 and pSG5 have been previously described [91].

### 4.2. Cells, Establishment of MCF7 Cells Stably Expressing ERβ1 or ERβ2, Whole Cell Binding Assay and Western Blot Analysis

The human breast adenocarcinoma cell line MCF7 was obtained from ATCC and maintained in Minimum Essential Medium (MEM) supplemented with 1 mg/L of insulin, 0.1 nM estradiol and 10% fetal bovine serum (FBS) [referred to as complete medium]. For stable expression of human ERβ1 or ERβ2, MCF7 cells were seeded in 10 cm dishes and transfected with the calcium phosphate co-precipitation method using 5 μg of pcDNA3.1-hERβ1 or pcDNA3.1-hERβ2 expression plasmids and 20 μg of pSG5. Then, 18 h after transfection, cells were washed with phosphate-buffered saline (PBS), fed with fresh medium and, 24 h later, were re-fed with medium containing geneticin (0.05 mg/mL of medium). Cells were fed with fresh geneticin-containing medium every 2–3 days, and colonies were isolated three weeks later and tested for expression of ERβ1 or ERβ2 by RT-qPCR. MCF7 clones were maintained in the same medium as MCF7 cells. Before experimental treatments, MCF7 cells (MCF7-WT) and their clones (MCF7-ERβ1 and MCF7-ERβ2) were cultured in MEM supplemented with 1 mg/L of insulin, 0.1 nM estradiol and 5% dextran-coated charcoal-stripped FBS (steroid-free medium) or 5% heat-inactivated FBS (HI-FBS), as indicated for each experiment. 

For the whole cell binding assay, cells cultured in steroid-free medium for 48 h were incubated with 0.1 nM [^3^H]estradiol, in the absence or presence of a 100-fold excess of unlabeled E2 for 1 h at 37 °C. Cells were washed, and specific cell-bound radioactivity was determined as previously described [92]. 

For western blot analysis of estrogen receptors, whole cell extracts from cells cultured in complete medium were analyzed by SDS-PAGE followed by immunoblotting using antibodies against ERα (D-12, sc-8005, Santa Cruz Biotechnology, Santa Cruz, CA, USA), ERβ1 (ab14021, Abcam Inc, Cambridge, UK) and ERβ2 (pBN1, [90]). For western blot analysis of Bax, Bcl-2 and cytochrome c, whole cell extracts from cells cultured in 5% HI-FBS-containing medium for 48 h and treated as indicated for 24 h were analyzed using antibodies against Bax (sc-493, Santa Cruz Biotechnology, USA), Bcl-2 (sc-509, Santa Cruz Biotechnology, USA), cytochrome c (sc-13560, Santa Cruz Biotechnology, USA) and actin (#MAB1501, Chemicon, USA). Proteins were visualized using ECL (GE healthcare). Image J v.1.53 software was used to quantify the optical densities on scanned films.

### 4.3. Crystal Violet Staining Assay, Flow Cytometry and Lactate Dehydrogenase (LDH) Assay 

Relative cell numbers were determined using the crystal violet staining assay as previously described [93]. Briefly, cells were cultured in 5% HI-FBS-containing medium for 24 h, treated as indicated for six days, fixed in ice-cold methanol, stained with 0.2% crystal violet (Sigma) and washed with tap water. The dye was extracted with acetic acid, and the absorbance was measured at 595 nm in a TECAN Safire 2 microplate reader. The difference in optical density at 595 and 690 nm (reference wavelength) was taken to measure the actual number of cells and was expressed relatively to that of cells treated with vehicle alone.

For flow cytometry, exponentially growing cells in 5% HI-FBS-containing medium were treated as indicated for 72 h. Attached and floating cells were collected together, fixed with ice-cold 50% ethanol in PBS and stored at 4 °C. Cells were washed, treated with 10 μg/mL RNAse A, stained with 50 μg/mL propidium iodide for 30 min at 4 °C and processed for flow cytometry in a FACSCalibur flow cytometer (Becton & Dickinson) with a minimum of 10,000 events collected for analysis with the ModFit LT program (Verity Software House).

Release of lactate dehydrogenase (LDH), a marker of cell death, was measured with the LDH Cytotoxicity Detection Kit (Takara Bio Inc., Otsu, Shiga, Japan) according to the manufacturer’s instructions. Briefly, cells were cultured in 5% HI-FBS-containing medium for 24 h and treated as indicated for four days. At the end of the treatment, the cell supernatant was incubated with an equal volume of LDH assay mixture for 30 min at 37 °C, and the absorbance was measured at 490 nm in a TECAN Safire 2 microplate reader. The difference in optical density at 490 and 690 nm (reference wavelength) was taken to measure the amount of released LDH. LDH in the cell pellet was extracting by incubation in culture medium containing 1% Triton X-100 at 37 °C for 2.5 h. Extracted LDH was measured as described for released LDH. Released LDH was expressed relatively to total (released and extracted from cell pellet) LDH. The deduced % released LDH was considered a measure of % dead cells.

### 4.4. RNA Isolation, Reverse Transcription and Real-Time PCR (qPCR)

Total RNA from cells cultured and treated as described for each experiment was extracted using the Trizol reagent (Life Technologies, Carlsbad, CA, USA) followed by purification using Qiagen RNeasy kit with on-column DNase treatment according to the manufacturer’s protocol (Qiagen, Venlo, Netherlands). Reverse transcription and qPCR were carried out as already described [94]. The comparative Ct method was used to calculate the relative gene expression by the formula 2^(−ΔCt)^. Expression levels of the genes of interest were normalized to the respective levels of GAPDH. The primer pairs for human genes used in this study are presented in Supplementary Table S13.

### 4.5. Illumina Array Analysis and Functional Interpretation

For microarray-based gene expression analysis, cells were cultured in 5% HI-FBS-containing medium for 24 h, treated as indicated for 24 h and used for total RNA isolation as described above. The Illumina HumanHT-12 V4.0 beadchip was used for gene expression analysis. Data import, pre-processing and normalization was performed with the R Bioconductor lumi package [95] (R v4.1.1). The raw dataset was imported as a LumiBatch object and was normalized with Variance Stabilization (VST), background adjustment and log transform. Batch effect correction was performed with the ComBat method implemented in the Bioconductor SVA package [96]. Differential expression analysis was performed with the Bioconductor limma package [97].

The BioInfoMiner web platform was used for the biological interpretation of the results [98], which performs functional enrichment and topological analysis of biomedical ontologies (Gene Ontology [99], Human Phenotype Ontology [100] and Mammalian Phenotype Ontology [101]) and pathways (Reactome pathway knowledgebase [102]). The tool applies various corrections to the ontologies (correction of gaps, annotation bias and other structural inconsistencies) and performs enrichment analysis to assess the over-representation of terms. It regroups the statistically significant terms into broader independent biological processes (systemic processes) and measures the functional contribution of the mapped genes according to their connectivity in the ontological network. Thus, it derives a comprehensive biological interpretation consisting of prioritized independent processes or pathways and prioritized genes ranked by their contribution to the systemic processes [103,104,105].

The data presented in this study have been deposited in ArrayExpress (accession number: E-MTAB-12549).

### 4.6. Statistics

The SPSS statistical package and the independent-samples *t*-test or one-way ANOVA were used, as indicated, to determine statistically significant differences (*p* < 0.05).

## Figures and Tables

**Table 1:** IC_50_ values of cell response to OHT or ICI as compared using one-way ANOVA. OHT, 4-hydroxytamoxifen; ICI, ICI182,780.

	Cell Line	OHT	ICI
IC_50_ (nM)	MCF7-WT	2.26 ± 0.90	0.76 ± 0.06
	MCF7-ERβ1	0.59 ± 0.09	0.55 ± 0.03
	MCF7-ERβ2	2.46 ± 0.35	0.96 ± 0.08
*p* value	MCF7-WT vs. MCF7-ERβ1	0.046	0.025
	MCF7-WT vs. MCF7-ERβ2	0.794	0.027
	MCF7-ERβ1 vs. MCF7-ERβ2	0.030	0.000

**Table 2:** IC_50_ values of cell response to ATRA as compared using one-way ANOVA. ATRA, all-trans retinoic acid.

	Cell Line	ATRA
IC_50_ (nM)	MCF7-WT	369 ± 62
	MCF7-ERβ1	89 ± 30
	MCF7-ERβ2	649 ± 80
*p* value	MCF7-WT vs. MCF7-ERβ1	0.013
	MCF7-WT vs. MCF7-ERβ2	0.011
	MCF7-ERβ1 vs. MCF7-ERβ2	0.000

**Table 3:** List of cell-death-related genes differentially expressed only in MCF7-WT cells.

Gene Symbol ^1^	Gene Name	Fold Change
RAI14	retinoic acid induced 14	1.73
IRF1	interferon regulatory factor 1	1.72
DHCR24	24-dehydrocholesterol reductase	1.71
HK2	hexokinase 2	1.59
SQSTM1	sequestosome 1	1.57
KRT4	keratin 4	1.56
**NKX3-1**	**NK3 homeobox 1**	**1.54**
DRAM1	DNA damage regulated autophagy modulator 1	1.53
S100A8	S100 calcium binding protein A8	0.49
TNFRSF11B	TNF receptor superfamily member 11b	0.50
KRT10	keratin 10	0.56
**BCL2**	**BCL2, apoptosis regulator**	**0.62**
CADM1	cell adhesion molecule 1	0.66

^1^ Genes are ordered according to their fold change, where values >1 denote upregulation and values <1 denote downregulation as compared to vehicle-treated cells. Hub genes are shown in bold.

**Table 4:** List of cell-death-related genes differentially expressed only in MCF7-ERβ1 cells.

Gene Symbol ^1^	Gene Name	Fold Change
AGR3	anterior gradient 3, protein disulphide isomerase family member	2.86
AKR1C3	aldo-keto reductase family 1 member C3	2.21
KRT13	keratin 13	2.21
**ALDH1A3**	**aldehyde dehydrogenase 1 family member A3**	**2.07**
MAG	myelin associated glycoprotein	2.05
NQO1	NAD(P)H quinone dehydrogenase 1	1.83
**HMGB2**	**high mobility group box 2**	**1.73**
SOX2	SRY-box 2	1.73
DOCK8	dedicator of cytokinesis 8	1.71
NDRG1	N-myc downstream regulated 1	1.69
ACAA2	acetyl-CoA acyltransferase 2	1.67
ACOX2	acyl-CoA oxidase 2	1.65
HMGB1	high mobility group box 1	1.63
**ZFP36L1**	**ZFP36 ring finger protein like 1**	**1.61**
ECT2	epithelial cell transforming 2	1.58
FGD3	FYVE, RhoGEF and PH domain containing 3	1.58
AGR2	anterior gradient 2, protein disulphide isomerase family member	1.55
**HIPK2**	**homeodomain interacting protein kinase 2**	**1.53**
BCL6	B-cell CLL/lymphoma 6	1.51
**PPARG**	**peroxisome proliferator activated receptor gamma**	**1.50**
ASCL1	achaete-scute family bHLH transcription factor 1	0.51
ID3	inhibitor of DNA binding 3, HLH protein	0.58
GPNMB	glycoprotein nmb	0.61
SGK3	serum/glucocorticoid regulated kinase family member 3	0.62
BAG3	BCL2 associated athanogene 3	0.64
ALKBH1	alkB homolog 1, histone H2A dioxygenase	0.65
WDR74	WD repeat domain 74	0.65
HIGD1A	HIG1 hypoxia inducible domain family member 1A	0.66

^1^ Genes are ordered according to their fold change, where values >1 denote upregulation and values <1 denote downregulation as compared to vehicle-treated cells. Hub genes are shown in bold.

**Table 5:** List of cell-death-related genes differentially expressed only in MCF7-ERβ2 cells.

Gene Symbol ^1^	Gene Name	Fold Change
**SIX4**	**SIX homeobox 4**	**1.86**
KRT80	keratin 80	1.60
GPR37L1	G protein-coupled receptor 37 like 1	1.60
BCL3	B-cell CLL/lymphoma 3	1.55
JAG2	jagged 2	1.53
PLK2	polo like kinase 2	1.53
IGFBP3	insulin like growth factor binding protein 3	1.52
ADARB1	adenosine deaminase, RNA specific B1	1.51
**CXCL12**	**C-X-C motif chemokine ligand 12**	**0.49**
INHBE	inhibin beta E subunit	0.50
IFIT2	interferon induced protein with tetratricopeptide repeats 2	0.60
GADD45B	growth arrest and DNA damage inducible beta	0.62
RPS7	ribosomal protein S7	0.64
ERN1	endoplasmic reticulum to nucleus signaling 1	0.64
FCMR	Fc fragment of IgM receptor	0.64
KRT6B	keratin 6B	0.64
IFIT3	interferon induced protein with tetratricopeptide repeats 3	0.65
TGFA	transforming growth factor alpha	0.66

^1^ Genes are ordered according to their fold change, where values >1 denote upregulation and values <1 denote downregulation as compared to vehicle-treated cells. Hub genes are shown in bold.

## Data Availability

The microarray data presented in this study have been deposited in ArrayExpress (accession number: E-MTAB-12549).

## Supplementary Materials

The following supporting information can be downloaded at: https://www.mdpi.com/article/10.3390/ijms24043747/s1.
