# Supplementary material for: ERβ1 Sensitizes and ERβ2 Desensitizes ERα-Positive Breast Cancer Cells to the Inhibitory Effects of Tamoxifen, Fulvestrant and Their Combination with All-Trans Retinoic Acid
Source: Int J Mol Sci. 2023 Feb 13;24(4):3747. doi: 10.3390/ijms24043747 (PMC9959521; doi:10.3390/ijms24043747)
Supplement: Supplementary file 1 [file ijms-24-03747-s001.zip › Manuscript_Meligova et al_Supplementary Files_proofread/Manuscript_Meligova et al_Supplementary Figure S1.pdf]

Figure S1. *Comparison of microarray-assessed and qPCR-assessed mRNA levels*

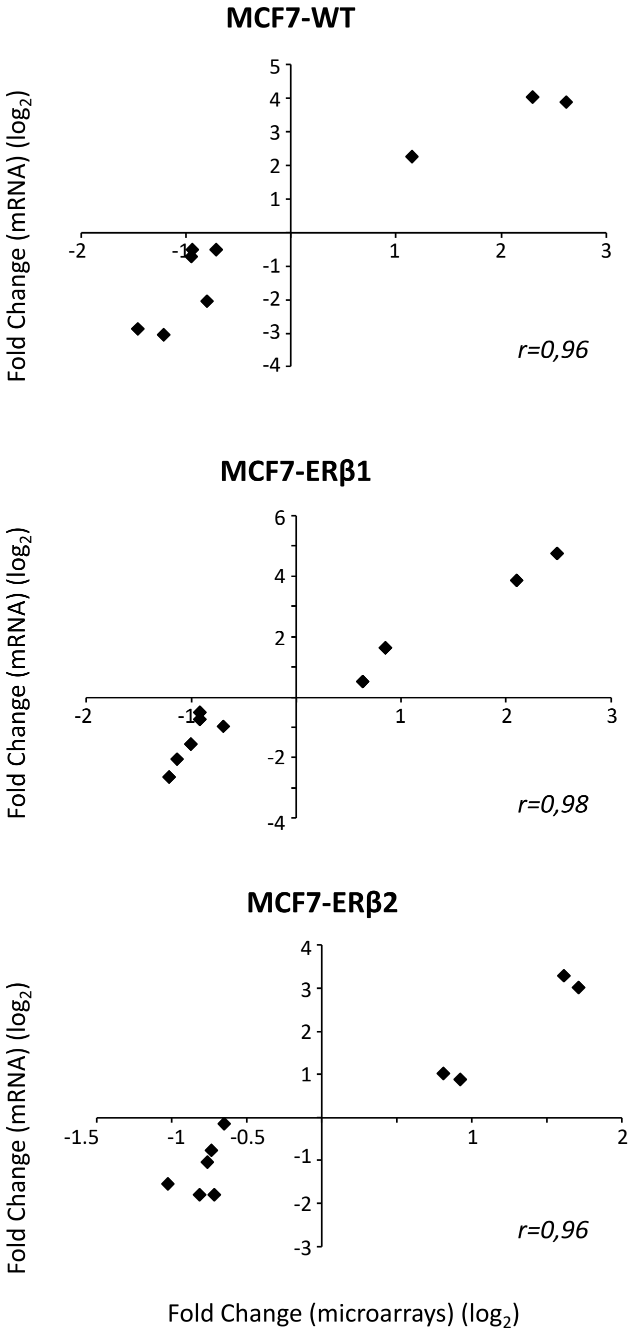

Scatter plots depicting the Pearson correlation (r) between changes in mRNA expression levels as assessed by qPCR (y-axis) and by microarray-based differential gene expression analysis (x-axis) of 10 randomly selected genes (BMP5, CAV1, ESR1, HOXA5, ID2, PGR, PLA2G10, SFN, TGFBI, THBS1) regulated by the combination of OHT and ATRA in MCF7-WT (upper panel), MCF7-ERβ1 (middle panel) and MCF7-ERβ2 cells (lower panel). Changes in mRNA expression levels were expressed as fold induction by the indicated treatment over vehicle (in log2 scale). OHT, 4-hydroxytamoxifen; ATRA, all-*trans* retinoic acid.
